# Characterizing Winter Wheat Germplasm for Fusarium Head Blight Resistance Under Accelerated Growth Conditions

**DOI:** 10.3389/fpls.2021.705006

**Published:** 2021-08-25

**Authors:** Mustafa Zakieh, David S. Gaikpa, Fernanda Leiva Sandoval, Marwan Alamrani, Tina Henriksson, Firuz Odilbekov, Aakash Chawade

**Affiliations:** ^1^Department of Plant Breeding, Swedish University of Agricultural Sciences, Lomma, Sweden; ^2^Lantmännen Lantbruk, Svalöv, Sweden

**Keywords:** Fusarium head blight, winter wheat, speed breeding, accelerated growth conditions, genome-wide association study, disease resistance

## Abstract

Fusarium head blight (FHB) is one of the economically important diseases of wheat as it causes severe yield loss and reduces grain quality. In winter wheat, due to its vernalization requirement, it takes an exceptionally long time for plants to reach the heading stage, thereby prolonging the time it takes for characterizing germplasm for FHB resistance. Therefore, in this work, we developed a protocol to evaluate winter wheat germplasm for FHB resistance under accelerated growth conditions. The protocol reduces the time required for plants to begin heading while avoiding any visible symptoms of stress on plants. The protocol was tested on 432 genotypes obtained from a breeding program and a genebank. The mean area under disease progress curve for FHB was 225.13 in the breeding set and 195.53 in the genebank set, indicating that the germplasm from the genebank set had higher resistance to FHB. In total, 10 quantitative trait loci (QTL) for FHB severity were identified by association mapping. Of these, nine QTL were identified in the combined set comprising both genebank and breeding sets, while two QTL each were identified in the breeding set and genebank set, respectively, when analyzed separately. Some QTLs overlapped between the three datasets. The results reveal that the protocol for FHB evaluation integrating accelerated growth conditions is an efficient approach for FHB resistance breeding in winter wheat and can be even applied to spring wheat after minor modifications.

## Introduction

Hexaploid winter wheat (*Triticum aestivum* L., 2n=6x=42, AABBDD) is an essential small-grain cereal crop grown for food and feed. In northern Europe, including Germany, wheat is the single most cultivated cereal crop where winter wheat is occupying the first place in production (Chawade et al., 2018). Studies examining global trends in wheat yield showed that with other major crops, wheat production must be doubled to meet the future demand to feed 10 billion people by the year 2050 (Ray et al., 2012; Hall and Richards, 2013; Ray et al., 2013). Current wheat production in the world is impacted by environmental factors, such as abiotic and biotic stresses and climate change. Meeting the 2050 demand is becoming increasingly dependent on the genetic improvement of new cultivars and developing novel techniques for agricultural practices. The investment in the development of new breeding methodologies for cultivar improvement emerged as one of the recommended strategies to tackle the 2050 challenges that are aiming to alleviate poverty, feed the 10 billion, and reduce greenhouse gas emissions (Searchinger et al., 2019). In northern Europe, wheat farming areas and yield trends have been increasing in the past decades (FAOSTAT, 2020), possibly driven by climate change where wheat productivity was positively correlated with warmer climates (Olesen and Bindi, 2002). However, factors that affect yield negatively in wheat are diseases, such as Septoria tritici blotch and Fusarium head blight (FHB; Chawade et al., 2018). FHB is one of the major diseases affecting winter (bread) wheat (Miedaner et al., 2010; Buerstmayr et al., 2020). The disease leads to reduced grain yield globally and is the second most serious disease affecting the wheat yield after leaf rust (Buerstmayr et al., 2020). FHB infected grains have poor quality as they contain mycotoxins which are harmful to humans and animal consumption (Schmolke et al., 2008; Buerstmayr et al., 2009; Berthiller et al., 2013; Nakagawa et al., 2017). Under humid and semi-humid conditions, FHB can severely impact wheat production and can lead to further losses due to increased accumulations of mycotoxins. This is of critical importance when considering the European Union maximum levels of mycotoxins allowed for cereals sold for food and feed production (European Union, 2020). Therefore, additional losses to FHB can be predicted mainly in rainy years. Previous experiences with severe FHB pandemic impacted farmers planting decisions as it was in the 1990s in some parts of the world (Ali and Vocke, 2009). Resistance to FHB in wheat can be dissected into five types that can be either evaluated independently or in combination with each other (Mesterhazy, 1995; Mesterhazy et al., 1999; Gong et al., 2020; Kumar et al., 2020). During the growth of plants, type I (initial infection of the florets) and type II (spread of the disease along the spike) have long been used for FHB resistance testing. In contrast, type III resistance (the accumulation of mycotoxins) can be evaluated during the development of FHB on the spikes and post-harvest. Type IV (kernel damage) and type V (reduction in yield) can be evaluated at the post-harvest stage. FHB resistance is quantitatively inherited, influenced by both additive and non-additive genetic effects (Venske et al., 2019; Ma et al., 2020; Ollier et al., 2020). Quantitative trait loci (QTL) mapping and genome-wide association studies (GWAS) are used extensively to identify QTLs for FHB resistance in wheat, for possible application in marker-assisted selection (Miedaner et al., 2019; Venske et al., 2019; Buerstmayr et al., 2020; Hu et al., 2020; Ollier et al., 2020).

Efforts to address FHB resistance through QTL mapping revealed so far the presence of 556 QTL spread across wheat genome (Steiner et al., 2017; Venske et al., 2019). The majority of the FHB resistance associated QTLs has been shown to add minor resistance effects to FHB in wheat (Schweiger et al., 2016; Fabre et al., 2020). However, a small subset of genes has been identified in FHB-mediated resistance (Venske et al., 2019; Fabre et al., 2020). The locus *Fhb1* found on chromosome 3BS has been long identified as a key player in mediating FHB resistance in wheat (Bai et al., 1999). More recent studies of the *Fhb1* revealed its role in harboring resistance to FHB by transforming *Arabidopsis* and FHB susceptible wheat cultivars with *Fhb1* locus (Rawat et al., 2016; Li et al., 2019; Su et al., 2019). Despite the conflicting results in terms of the mechanisms on how *Fhb1* is mediating the resistance, cloning the locus validated its strong association in enhancing the resistance in the susceptible genotypes (Rawat et al., 2016; Li et al., 2019; Su et al., 2019). Driven by its role in FHB resistance, several studies were carried out to identify the presence of *Fhb1* locus in the germplasms adapted in breeding programs for many regions in the world (Liu and Anderson, 2003; Wang et al., 2017; Zhu et al., 2020). However, so far, studies have demonstrated a low frequency of *Fhb1* in their germplasms (Hao et al., 2020; Zhu et al., 2020). Interestingly, *Fhb1* is reportedly the only resistance QTL found in many new European wheat cultivars exhibiting high resistance levels (Hao et al., 2020).

The winter wheat growth cycle is relatively longer compared to the spring cereal crops, as winter wheat requires a vernalization period of up to 12weeks to initiate the reproductive growth period (Ferrie and Polowick, 2020). Thus, up to two generations of winter wheat a year can be achieved in greenhouse growth conditions provided there is infrastructure available for vernalization (Ferrie and Polowick, 2020). Reducing the growth cycle is of paramount importance in increasing the genetic gain of the crops (Cobb et al., 2019). While the vernalization period of winter wheat is a limiting factor in shortening its life cycle (Voss-Fels et al., 2019), speeding up winter wheat life cycle can be achieved by optimizing post-vernalization growth conditions. The speed breeding (SB) technique in spring crops is shown to accelerate the growth and development of plants resulting in considerably shortening the time from sowing to harvest (Ghosh et al., 2018; Watson et al., 2018; Hickey et al., 2019). SB can be achieved by using an artificially prolonged light period, increased daylight intensity where light quality can be controlled (Ghosh et al., 2018; Watson et al., 2018). Under SB conditions, up to six generations of spring wheat and spring barley can be completed in 1year (Hickey et al., 2019). SB protocols were also developed for other plant species, including peanuts, chickpea, oats, and quinoa (Hickey et al., 2019).

Growing plants in controlled environments can greatly reduce the environmental variation associated with field trials and allow the possibility of several screening per year without being limited to one season in the field (Riaz et al., 2016). Aspects plant development under continuous light conditions SB must be in the direction of enhancing the growth rate without negatively affecting the steps undertaken for the evolution of disease resistance. The phenotypic characterization of leaf rust resistance in spring wheat plants grown under artificial conditions has been shown to give similar results to those in field trials (Riaz et al., 2016). In winter wheat, and regardless of the photoperiodism and vernalization, the developmental rate of the plants has been shown to be positively promoted in continuous light setting made with a light spectrum from combining different fluorescent light lamps grown constantly at 20°C (Sysoeva et al., 2010). Increased photosynthetic rate of several crops including wheat has been observed in long-day conditions leading to increased dry matter accumulation where the partitioning of the dry matter appears to be undisrupted by the continues light in wheat (Sysoeva et al., 2010). More recent studies have revealed the even though some physiological disorders in wheat plants have been observed when grown under continuous light (Sysoeva et al., 2010), other studies indicated suitability of SB for wheat (Ghosh et al., 2018). The light settings provided by LED light spots giving light spectrum of blue, red, and far-red with photosynthetic photon flux density between 540 and 500μmolm^−2^ s^−1^ for 22h/day have been shown to be suitable in SB of spring wheat and barley plants (Ghosh et al., 2018). Winter wheat may slightly differ in its light responses compared to spring wheat. Therefore, light settings must be adjusted (photoperiod, composition, and intensities) so light injury reflected by symptoms, such as leaf chlorosis, are not visible.

This study aimed to develop a protocol to combine accelerated growth conditions under SB with the evaluation of FHB resistance in winter wheat plants. The developed protocol was tested using two different sets of germplasm obtained from the breeding program and the genebank. The germplasm phenotypic characterization was later used for GWAS to identify QTL in the studied germplasm. The developed protocol and the results from the germplasm characterization are presented.

## Materials and Methods

### Plant Material

The plant material used in this work included winter wheat germplasm from two different sources. The first group of winter wheat genotypes was made up of 181 genotypes of highly diverse plant materials that included landraces and old cultivars (genebank set) obtained from the Nordic Genetic Resource Center (Nordgen). The second source of the plant material consisted of 338 genotypes (breeding set) provided by the Swedish agricultural cooperative (Lantmännen Lantbruk, Svalöv, Sweden).

### Plant Growth Conditions

#### Germination

This work was conducted in the biotron, a facility with controlled-climate chambers at the Swedish University of Agricultural Sciences (SLU) in Alnarp, Sweden. Several seeds of each genotype were planted in 8×8×8cm plastic pots filled with peat soil from Emmaljunga Torvmull AB, Sweden. The pots were arranged using the augmented block design described under the experimental design section. The pots were watered as required, and the seeds were left to germinate for 5days. During the seed germination period, day-length parameters were adjusted at a light intensity (LI) of 250μmolm^−2^ s^−1^ for 8h at °C 22, night 16h of darkness with at 20°C while keeping relative humidity (RH) of 50%. After successful germination, plants were thinned and only one plant was allowed to grow in each pot.

#### Vernalization

Seedlings were vernalized by growing under short-day conditions of 8/16h day/night regime with the temperature of 3°C and LI of 250μmolm^−2^ s^−1^. At this intensity, vernalization light source, wavelength composition, and individual wavelength intensities are described under accelerated growth conditions. RH was 80% for 8–9weeks (approximately 60days).

#### Acclimatization

After vernalization, plants were allowed to acclimatize to the upcoming vegetative growth period. This included a period of gradual change in growth conditions for 6days (Table 1). The temperature was set to increase per day by 3–4°C and day-length by 2–3h. LI was increased to 400μmolm^−2^ s^−1^ on the second day and was left unchanged throughout the acclimatization period. RH was gradually lowered to reach 50% at the end of the acclimatization (Table 1).

**Table 1:** Growth conditions for acclimatization of vernalized winter wheat plants to the growth conditions of accelerated growth.

Days after vernalization	Temp °C	Day/Night (Hours)	Light intensity μmol m^−2^ s^−1^	Relative humidity %
1	3	8/16	250	80
2	6	11/13	400	80
3	9	14/10	400	80
4	12	17/7	400	80
5	15	20/4	400	50
6	18	22/2	400	50
7	22	22/2	400	50

#### Accelerated Growth Conditions

At the end of the acclimatization period, the plants were allowed to grow for 32days under the same conditions as on the last acclimatization day (Table 1). The lighting source was LED lights model RX30 grow lights (Heliospectra AB, Gothenburg, Sweden). The LED grow lights provided nine individually controlled wavelengths ranging from 380nm (UVA) to 735nm (far-red) and white light. Wavelengths 380, 400, 420, and 450 were set to radiate at 480μmolm^−2^ s^−1^ intensity. Meanwhile, the remaining wavelengths that included 530, 620, 660, 735, and the white light were adjusted with high intensity at 960μmolm^−2^ s^−1^. Sensor-feedback-based lighting continuously adjusted at the level of the plant canopy was set to give 400μmolm^−2^ s^−1^ intensity from the light source for 22h. The temperature throughout the extended long day was constantly maintained at 22°C following the speed breeding protocol published earlier (Ghosh et al., 2018). Due to the rapid nature of plant growth under the extended long-day conditions, a schedule of daily watering and weekly fertilization was followed. Initially, a mix of high phosphate and high nitrogen soluble fertilizer SW-BOUYANT 7-1-5+Mikro+KH_2_PO_4_ was added 3days post-acclimatization (dpa). High nitrogen fertilizer was added at 10 dpa followed by high potassium soluble fertilizer Yara Tera Kristalon NPK 12-5-30 with S, and micro was added twice at 15 and 20 dpa.

#### Inoculum Preparation for Fusarium Head Blight

Isolates belonging to *Fusarium* species *F. graminearum* and *F. culmorum* provided by the plant breeding company Lantmännen Lantbruk were used in the preparation of the inoculum. These included six isolates of *F. graminearum* and three isolates of *F. culmorum*. Using a large number of isolates was intended to identify germplasm with broad resistance to various *Fusarium* species. The isolates were cultured on the weak Spezieller Nahrstoffarmer agar media (Leslie and Summerell, 2006). The cultures were incubated at 24°C for 4days, followed by near ultra-violet UV radiation for 10h to promote macroconidial formation. Following the UV light treatment, the cultures were moved back to incubate for another 3–4days at 24°C before collecting macroconidial spores for the inoculum preparation by pouring water on the surface of the cultures and scarping using a spatula. The surfactant Tween^®^20 0.002% (v/v) was added to the final suspension containing the spore concentration of 5×10^5^ spore/ml.

#### FHB Infection Conditions

Upon completing ear emergence and the emergence of anthers, approximately 33 dpa plants were moved to grow under a long-day regime with 16/8h day/night in the greenhouse chamber. RH was adjusted to 60%, and the temperature was maintained at 24°C. The new growth conditions were intended to allow the plants to continue growing for 24days without accelerated growth until physiological maturity. Daily watering and weekly fertilization were carried out at this stage. Plants at 75% heading were spray-inoculated once, and inoculated plants were incubated at a high RH of 90% for 48h while keeping other growth parameters unchanged. At the end of this incubation period, RH was lowered to 60%, and plants were allowed to grow until the end of the 24day period.

**Figure 1:**
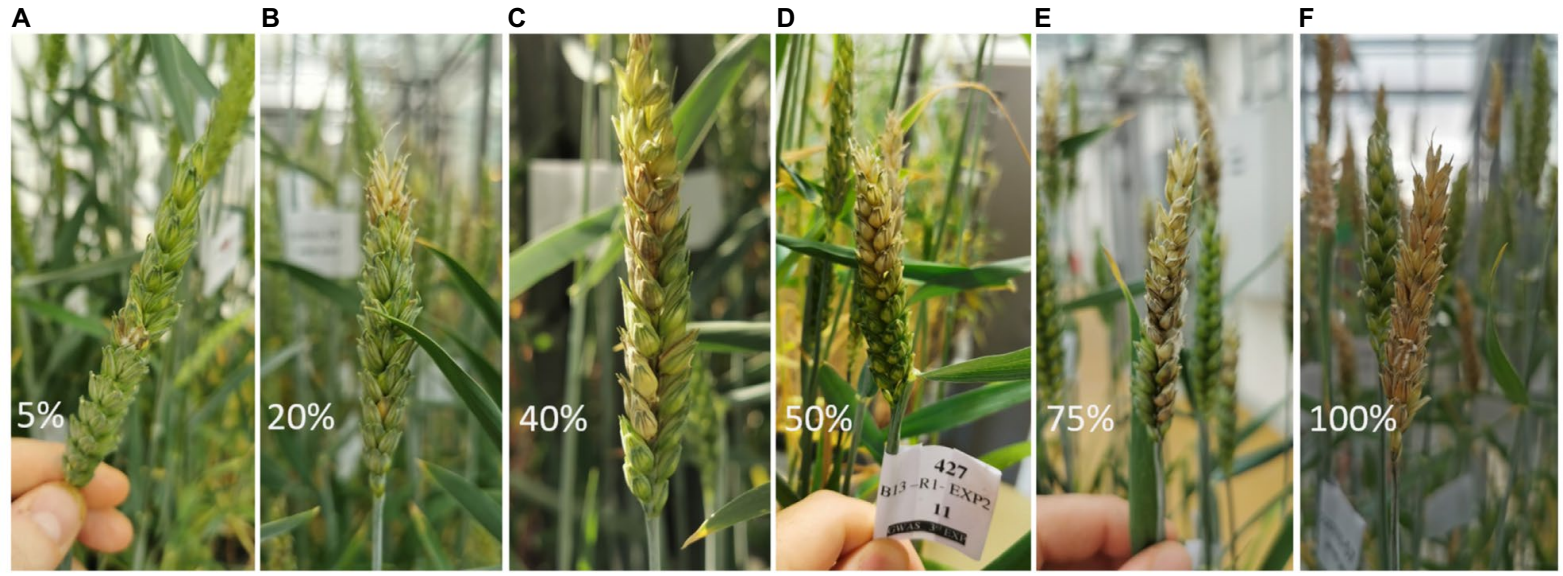
Scale for Fusarium head blight (FHB) severity scoring on winter wheat spike. Rating of disease severity ranged from **(A)** 5 to **(F)** 100%. FHB infection can be continues **(B, D)** or disconnected **(C, E)** on a spike. Scoring was based on the proportion of total infected spikelets to the total numbers of spikelets.

The visual assessment of FHB disease severity on the spikes was carried out at 6, 8, 10, and 12-days post-inoculation (dpi). Generally, visual symptoms, such as bleached, yellowish or discolored, and stunted spikes, indicate the development of FHB on the ears. Disease spread was evaluated as percentage infection ranging between 5% (most resistant phenotypes) and 100% (most susceptible phenotype). The percentage rating scoring was based on the relative number of infected spikelets to the total number of spikelets per spike on the main tiller (Stack and McMullen, 1998) with an adjustment of the scoring method. Unlike the visual assessment of disease spread of FHB type II resistance, the current scoring method relied on assessing the disease severity in relation to all infected spikelets on the ear regardless of the symptom continuity. Figure 1 shows the scale used for the visual assessment of FHB severity. Discontinued spread of the disease (symptoms are located distantly on the same spike and separated by spikelets that show no visual FHB infection symptoms) is taken together to represent the total severity on the spike (Figures 1C,E).

The genotypic variation in heading and flowering represents a challenge that may affect the uniformity of FHB development on a large and diverse number of artificially inoculated plants. Additionally, certain genotypes may require longer periods of vernalization to promote heading and subsequently flowering leading to the inoculated plants at earlier stage for those genotypes compared to the rest of the genotypes in the germplasm. In order to limit the bias in the downstream analysis of FHB resistance, germplasm genotypes that showed 0% infection phenotype (absence of infection symptoms) in the material were discarded together with genotypes that have not reached heading at the time of inoculation. Only genotypes that scored varying FHB symptoms that ranged between 5 and 100% were included in the analysis.

#### Harvest

Watering was discontinued 21days after FHB infection conditions while keeping all other growing conditions unchanged. RH was lowered to 40% 24days after reproductive growth in the greenhouse and the plants were left to mature. Spikes were harvested approximately 30days after FHB infection conditions.

### Flag Leaf Area, Spike Length, and Spike Width Measurements

During the reproductive growth period, flag leaf area (FLA) was measured for each genotype using LI-3000C Portable Leaf Area Meter. Spike length (SL) and spike width (SW) were estimated using a digital Vernier caliper scale. In order to avoid bias in SW (thickness of the spike), width measurement was always performed at the third lower spikelet.

### Heading Time and Anther Extrusion

Heading time (HT) was taken depending on the emergence of 75% of the spikes out of the sheath of the flag leaf at three time points recorded every third day consecutively. Spikes were categorized according to the three HTs as early (HT1), medium (HT2), and late (HT3). Anther extrusion was recorded at two time points with 2days difference and was recorded as early (AE1) and late (AE2).

### Experimental Design

Four replicates each of genebank and breeding sets were arranged in an augmented block design developed using the package Agricolae in R (De Mendiburu, 2014). The design included four checks of winter wheat cultivars per block, namely, Nimbus, Stigg, Norin, and Julius. According to this design, 11 blocks per replicate were assigned for the breeding set and six blocks per replicate for the genebank set.

### Phenotypic Analyses

Unadjusted means of cultivars within the augmented design of each replicate were filtered and removed for the cultivars that gave a percentage of 0%. Phenotypic data were analyzed in two steps. First, the checks in each augmented block were used to adjust the means for each trait per experiment/replicate using the Agricolae R package (De Mendiburu, 2014) based on the following model:

y_il=u+G_il+B_l+ε_il,

where, y_il is the adjusted means of the ith wheat genotype in the lth block, u is the general mean value, G_il is the effect of the ith wheat genotype in the lth block, B_l is the lth block effect, and ε_il is the residual. For FHB severity, the area under disease progress curve (AUDPC) was estimated from the adjusted means of the four disease ratings for each experiment. In the second step, the adjusted means were used to calculate the best linear unbiased estimates (BLUEs) following the randomized complete block design option in META-R 6.04 (Alvarado et al., 2015) based on the model:

y_ijm=u+S_j+G_ijm+R_m+ε_ijm,

where, y_ijm is the BLUE of the ith wheat genotype from the jth source/population in mth replicate, u is the general mean value, S_j is the effect of the jth source of material, G_ijm is effect of the ith wheat genotype in the mth replicate, R_m is the mth replicate effect, and ε_ijm is the residual effect. The source of wheat genotypes, S_j, was treated as the grouping factor.

### Genotyping and Genome-Wide Association Studies

The genebank set was genotyped previously using a 20K SNP marker array as described by Odilbekov et al. (2019). While the breeding set was genotyped using the 25K SNP chip by TraitGenetics GmbH, Germany.^1^ Markers with ≥20% missing values were removed. The remaining missing values were imputed by setting SNP.impute=“Major” in Genome Association and Integrated Prediction Tool (GAPIT) 3.0 R package (Lipka et al., 2012). After the quality check, 432 lines (breeding set: 272 and genebank set: 160) and 10,328 SNP markers were left for all genome-based analyses.

Seven models were used for the GWAS: general linear model (Pritchard et al., 2000), mixed linear model (Yu et al., 2005), compressed MLM (Zhang et al., 2010), settlement of MLM under progressively exclusive relationship (Wang et al., 2014), multiple locus linear mixed-model (Segura et al., 2012), fixed and random model circulating probability unification (Liu et al., 2016), and Bayesian-information and linkage-disequilibrium iteratively nested keyway (Huang et al., 2018) implemented in R package GAPIT version 3.0 (Lipka et al., 2012). GLM, MLM, CMLM, and SUPER are single locus GWAS models while MLMM, FarmCPU, and Blink are multiple loci GWAS models (described in detail by the respective authors cited above). The kinship (K) and top 5 to 10 principal components (PCs) were used depending on the model and trait, to control familial relatedness and possible population structure following the settings in GAPIT 3.0 (Lipka et al., 2012).

## Results

### Accelerated Growth With FHB Protocol for Winter Wheat

**Figure 2:**
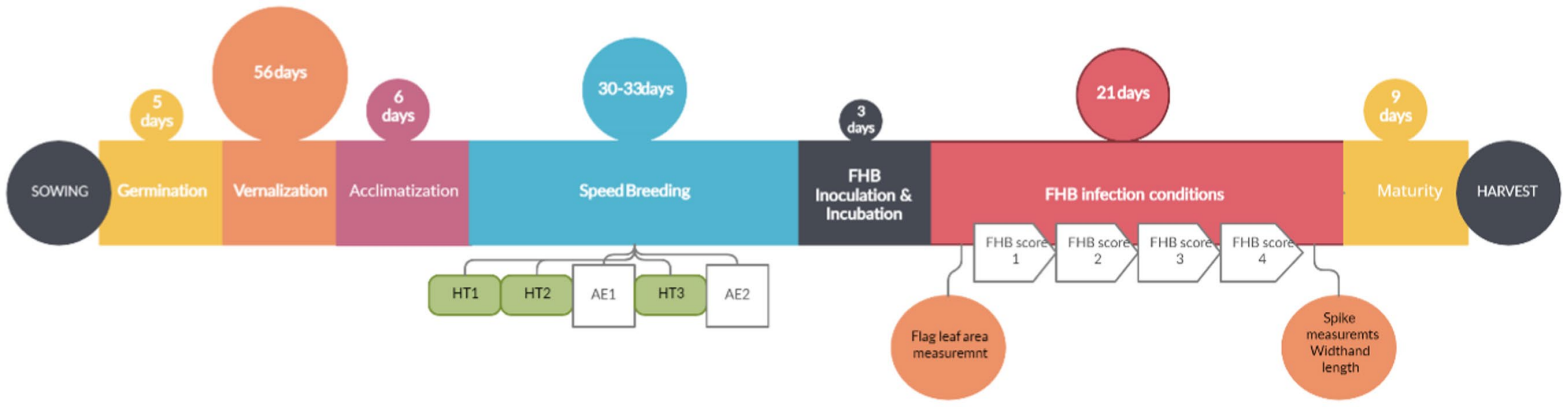
Schematic overview of the AGFHB protocol for FHB evaluation in winter wheat. Time points for the three time points of heading, heading time HT1 to HT3. Anther extrusion times AE1 and AE2. FHB scoring time points FHB score 1 to 4.

**Figure 3:**
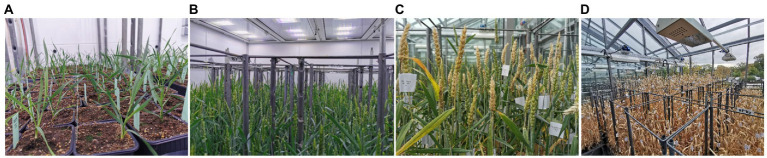
The rapid development of winter wheat plants under accelerated growth conditions. **(A)** first day post-acclimatization (dpa); **(B)** 31 dpa end of accelerated growth; **(C)** winter wheat ears showing FHB symptoms; and **(D)** maturity.

The protocol for winter wheat using accelerated growth for the evaluation of FHB resistance (AGFHB) consisted of three major growth periods, namely, (a) the pre-accelerated growth period when the plants were allowed to germinate and vernalize under optimal growth conditions; (b) the accelerated growth period when the plant growth was fast-tracked; and (c) the FHB infection period when the plants were grown in conditions optimal for FHB infection and maturity (Figure 2). The pre-accelerated growth consisted of germination, vernalization, and acclimatization phases. Germination was promoted for 5days followed by vernalization for 56days. Thereafter, to acclimatize the plants for the upcoming stage, the growth conditions were gradually changed over a period of 6days. During this time, the temperature was gradually increased from 3°C to 22°C, day-length was gradually increased from 8h to 22h, and light intensity was increased from 250 to 400μmolm^−2^ s^−1^ while RH was decreased from 80 to 50%. After that, accelerated growth conditions allowed plants to rapidly reach the reproductive phase within 30–33days while limiting any visible symptoms of plant stress. At this stage, scoring for heading time, anthesis time, and FLA was performed. Thereafter, FHB infection conditions were introduced to promote FHB infection. Plants were thereafter allowed to mature before harvesting (Figure 3). The entire protocol took between 120 and 130days depending on the genotype.

### Evaluation of Agronomic Traits of Germplasm

The AGFHB protocol was used for the evaluation of a total of 519 genotypes consisting of 181 genotypes in the genebank set and 338 genotypes in the breeding set. At the time of the FHB inoculation, 88 and 90% of the plants completed 75% heading of their spikes intended for FHB resistance evaluation in the breeding and genebank sets, respectively. With regard to flowering, 67 and 88% of the plants reached anthesis in the breeding and genebank sets, respectively. As previously stated, genotypes that did not reach the stage of 75% heading at inoculation time were discarded from the following FHB severity scoring together with genotypes that exhibited no visual disease development on the ears.

**Figure 4:**
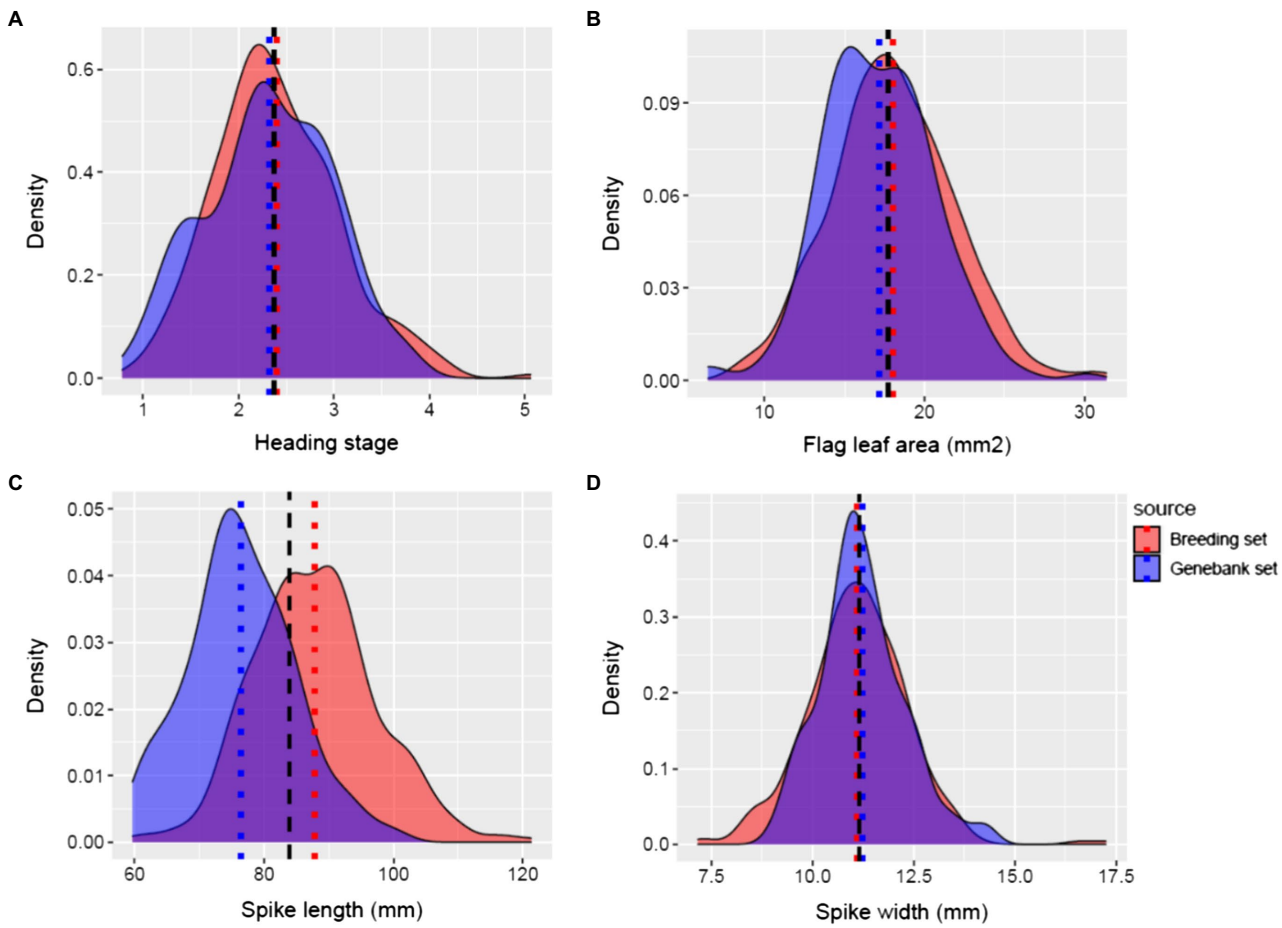
Phenotypic distribution of **(A)** heading stage, **(B)** flag leaf area, **(C)** spike length, and **(D)** spike width. Breeding set (red) and genebank set (blue). The black dashed line represents the overall mean for combined genotypes from both breeding set and genebank set.

Best linear unbiased estimates of measured agronomic traits of genebank and breeding sets showed that the mean heading stage was similar in both source populations (Figure 4A). The mean FLA of the breeding set was 18.02mm^2^ (*s*=3.87), while for the genebank set, it was 17.15mm^2^ (*s*=3.50; Figure 4B). Thus, the mean FLA of the genebank set was smaller compared to the breeding set. The mean SL in the genebank set was 76.44mm (*s*=8.29), while in the breeding set was 87.82mm (*s*=9.47; Figure 4C). SL was smaller in the genebank set compared to the breeding set. The mean SW in the genebank set was 11.23mm (*s*=1.05), while in the breeding set was 11.10mm (*s*=1.25; Figure 4D).

### FHB Evaluation

**Figure 5:**
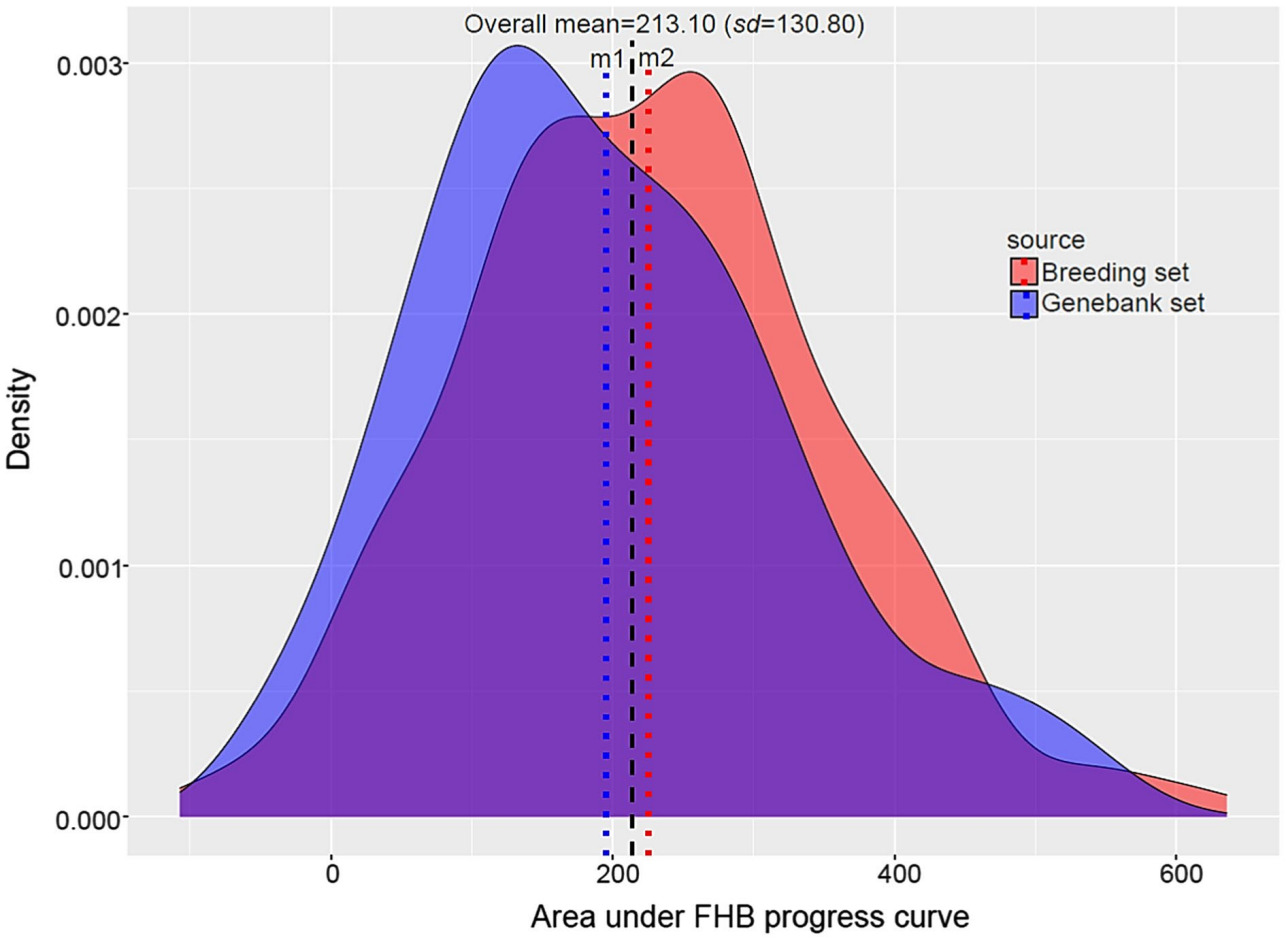
Histogram of the area under disease progress curve (AUDPC) for FHB in the wheat genotypes collected from two sources. m1 and m2 represent the mean AUDPC for FHB in genebank set and breeding set, respectively.

Fusarium head blight progression was evaluated at four time points and recorded by visually assessing the percentage of FHB on the main tiller spike of each plant. The BLUEs of the area under the FHB progress curve used for our GWAS showed approximately normal phenotypic distribution with an overall mean of 213.10 (*s*=130.80). The average AUDPC was 225.13 (*s*=129.98) for breeding set and 195.53 (*s*=130.44) for genebank set (Figure 5). The correlation between FHB severity (AUDPC) and the five agronomic traits was weak and non-significant in most instances (Supplementary Figure 1). The correlation between heading and anthesis was moderate and highly significant (*r*=0.51, *p*<0.001). We found highly significant genotypic variances (*p*<0.0001) and moderate to high broad-sense heritabilities, depending on the trait and the source of genotypes (Supplementary Table 1). Broad-sense heritability for FHB based on replication in time and space was 0.55 in the combined set, 0.57 in the genebank set, and 0.53 in the breeding set.

To further evaluate the FHB severity estimates from this work, comparison was done with FHB scores from a previous field trial from 2019 conducted by the breeding company Lantmännen Lantbruk. The FHB scores from the field trial were collected in the scale of 1–8. From the breeding set, 275 genotypes were found to be common in the two datasets. A spearman correlation of 0.24 was observed in the FHB scores between the two datasets. When the genotypes were grouped as resistant (FHB scores 1–3) and susceptible (FHB scores 6–8) a statistically significant difference (*p*<0.0001) was observed between the two groups for mean FHB estimates obtained under controlled conditions.

### Genome-Wide Association Studies

**Figure 6:**
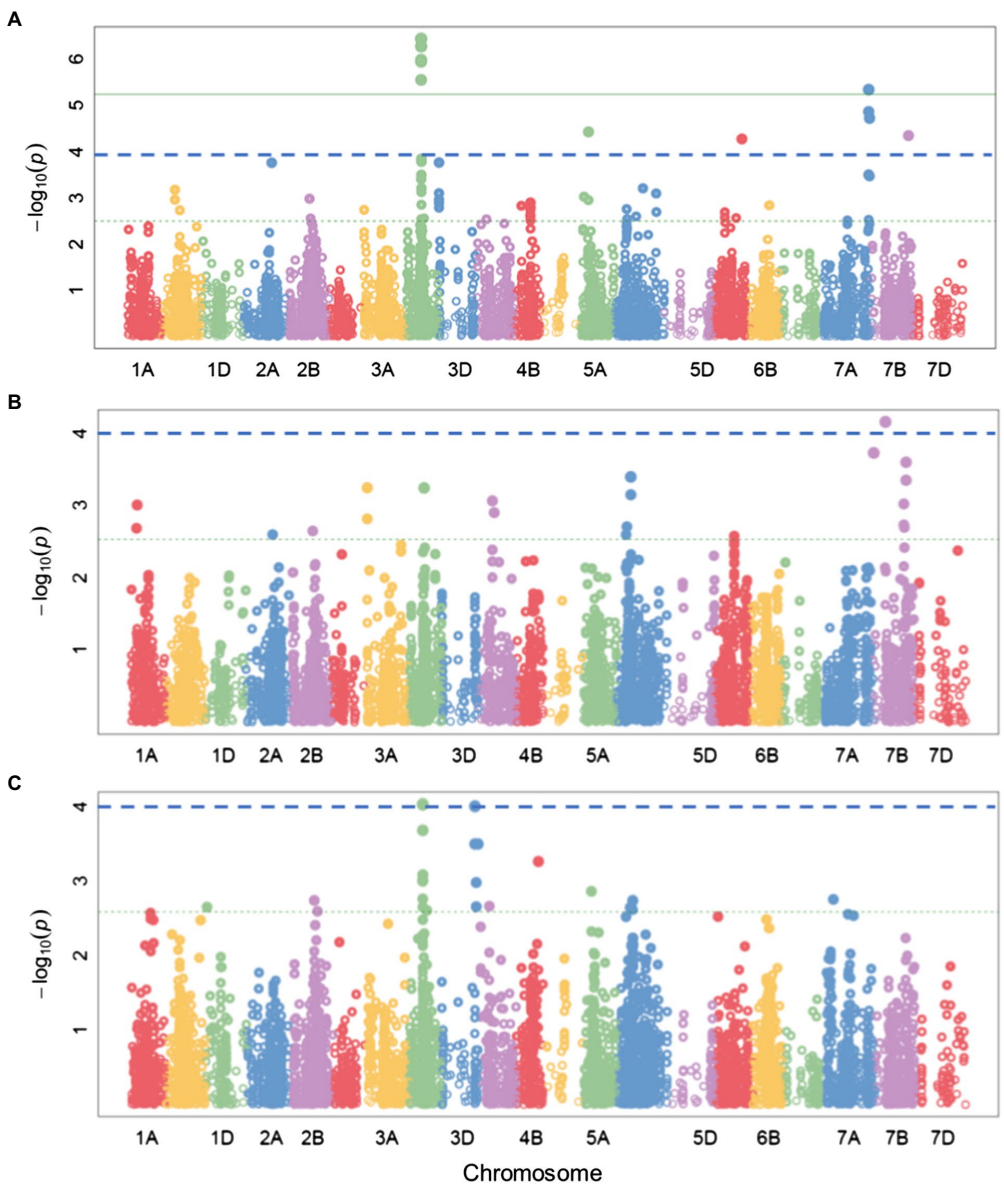
Manhattan plots of AUDPC (FHB severity) identified with SUPER **(A)** combined set; **(B)** breeding set; and **(C)** genebank set. Green continuous and blue dashed horizontal lines represent Bonferroni corrected threshold at *α*=0.05 and exploratory threshold at *p*= 0.0001, respectively.

The multi-model GWAS detected 12 significant SNPs associated with nine QTLs for FHB severity (*p*≤0.0001) in the combined dataset (*N*=432). Four QTLs were co-detected by at least two GWAS models (*p*≤0.0001, Table 2). Three SNPs, wsnp_Ex_c34975_43204180, Kukri_c18009_398 (chromosome, chr. 3B), and RAC875_c12733_1509 (chr. 7A), were detected above the Bonferroni corrected threshold by SUPER and Blink models (*α*=0.05, Figure 6). The SNPs associated with the QTLs on chr. 3B (qtlfhb4) had the largest marker effects (Table 2). The majority of the SNPs detected in the combined dataset as well as within the breeding set (*N*=272) and genebank set (*N*=160) for the resistance against FHB severity was located on the sub-genome B (Table 2; Figure 6; Supplementary Table 2). Additionally, we found several significant SNPs for the five agronomic traits (*p*=0.0001, Supplementary Tables 3 and 4). At least two GWAS models simultaneously detected 21, 5, 3, 14, and 2 markers for heading, anthesis, SW, SL, and FLA, respectively, in the 432 wheat lines (Supplementary Table 3). A few SNPs were associated with common QTLs between these traits (Supplementary Table 3). Two QTLs on chr. 3B and 6A were common between FHB severity and heading stage (Table 2; Supplementary Table 3). At *p*=0.0001, for all traits, we found more QTLs using lines from breeding set and genebank set combined (*N*=432) than for lines from within each source population alone (Table 2; Supplementary Tables 2–4; Figure 6). As a result, we lowered the significant threshold to *p*=0.001 [−log(*P*)=3] for the GWAS within each source population (Supplementary Tables 2–4).

**Table 2:** Quantitative trait loci (QTLs) detected by seven GWAS models at *p*=0.0001 (LOD≥4) for FHB severity in winter wheat from combined (CS), breeding (BS), and genebank (GS) sets.

QTL	Marker	Chr.	Position (cM)	FAF	Effect^b^	Model(s)	Set
SLUfhbchr1B.1	BS00021877_51	1B	154.58	0.06	NA	Blink	Combined
SLUfhbchr2A.2	BobWhite_c16923_64	2A	125.33	0.06	NA	Blink; (SUPER)^*^	Combined
SLUfhbchr3A.3	Kukri_rep_c89183_282	3A	15.05	0.64	27.84 to 28.10	GLM, CMLM	Combined
SLUfhbchr3B.4	wsnp_Ex_c34975_43204180^a^	3B	67.45	0.95(CS), 0.94 (BS), 0.97(GS)	65.78 to 82.47	GLM, MLM, CMLM, SUPER, MLMM, FarmCPU, Blink	Combined, Breeding, Genebank
	Kukri_c18009_398^a^	3B	67.67	0.95	78.20 to 80.15	GLM, MLM, CMLM, SUPER	Combined
	wsnp_Ex_c5378_9505533	3B	68.71	0.94	NA	SUPER	Combined
SLUfhbchr3D.5a	RFL_Contig4591_1759	3D	0.00	0.94	51.94 to 54.69^*^	MLMM; (GLM, MLM, CLM, SUPER, Blink)^*^	Combined
	RAC875_rep_c115090_51	3D	0.00	0.02	NA	Blink	Breeding
SLUfhbchr3D.5b	JD_c7714_954	3D	143.01	0.04	NA	Blink, SUPER	Genebank
SLUfhbchr5A.6	RAC875_rep_c106118_339	5A	39.02	0.03	−31.55 to −29.40	GLM, MLM, SUPER, MLMM	Combined
SLUfhbch6A.7	Tdurum_contig46670_911	6A	128.26	0.96	NA	SUPER	Combined
SLUfhbchr7A.8	Kukri_c11530_92	7A	232.11	0.84	44.1	CMLM, SUPER, MLMM	Combined
	RAC875_c12733_1509^a^	7A	228.37	0.83	40.41 to 45.14	GLM, MLM, CMLM, SUPER, MLMM, FarmCPU, Blink	Combined
SLUfhbchr7B.9	wsnp_Ex_c351_689415	7B	143.23	0.02	NA	Blink, SUPER	Breeding
	RAC875_c8752_1079	7B	158.98	0.84	39.97^*^	SUPER; (CMLM)^*^	Combined

*Chr., chromosome; FAF, favorable allele frequencies*;

* *also detected by these models at p=0.0002*.

a detected above Bonferroni corrected threshold (α=0.05).

b marker effects are estimated for only GLM, MLM, and CMLM; and FarmCPU in GAPIT (Lipka et al., 2012).

## Discussion

Developing and implementing new techniques to accelerate wheat genetic gains are essential to achieve the goal of feeding 10 billion people by 2050. Crop genetic gain for disease resistance can be accelerated by reducing generation time and increasing selection intensity. Increasing the genetic gain will not only contribute to increasing the genetic diversity for resistance but will also enable faster introgressions and selection of resistance genes in wheat. It takes up to 10years to develop a new winter wheat cultivar; thus, accelerating this process by increasing the number of generations per year can contribute to the genetic gain of wheat when breeding for yield, climate resilience, and biotic and abiotic stresses. SB is a technique that utilizes affordable growing equipment under greenhouse conditions to shorten generation time in plants. This technique was shown to be effective in several crops, including spring wheat, spring barley, chickpea, oat, quinoa, peanut, and amaranth (Watson et al., 2018; Hickey et al., 2019).

In this work, we developed a protocol to integrate accelerated growth with FHB resistance screening, followed by association mapping. Previously, SB was used to introgress resistance to four diseases in barley in a modified backcross strategy and plants were evaluated and selected based on disease resistance under accelerated growth conditions and later in field trials (Hickey et al., 2017). The protocol proposed in this work allows accelerated growth while avoiding any visible stress symptoms on plants, which is necessary to be able to screen for disease resistance. While the plants are grown under accelerated growth conditions until heading, the growth conditions are changed to regular growth conditions prior to inoculation for FHB which allows the plants to stabilize prior to FHB infection. This provides an advantage of reduced time to reach heading while obtaining disease resistance scores based on plants grown under regular growth conditions. It could be postulated though that there are certain molecular responses in plants activated due to the accelerated growth which continues to remain active even after plants receive regular growth conditions during FHB infection. Further research would be required to fully understand and unravel such responses. It was earlier shown that the most resistant wheat line consistently expressed highest resistance for FHB severity and deoxynivalenol under both greenhouse and field conditions (Kang et al., 2011) suggesting that evaluating plants for resistance to FHB under controlled conditions can accelerate resistance breeding for FHB. Previous studies on winter wheat grown under SB conditions reported 105.4±1.7days are needed to reach flowering of winter wheat (Ghosh et al., 2018). The current protocol shortens the period required from sowing to anthesis of the plants to 97–100days. Moreover, while FHB resistance is screened for in a large number of genotypes, the whole period required from seed to seed is achieved within a time frame of 120–130days. The current protocol enables the evaluation of FHB resistance in three consequent generations of winter wheat per year compared to two generations under regular growth conditions in a greenhouse.

Previous work on comparing measurements taken to evaluate leaf rust resistance in spring wheat grown in controlled environment with continuous light and field conditions showed that the source of variation for the resistance was greatly genotypic (Riaz et al., 2016). The evaluated resistance to leaf rust under continuous light was correlated to that in the field in a panel of diverse cultivars of spring wheat (Riaz et al., 2016). Despite the dissimilarities in terms of growth conditions between the current protocol and field trials, the variations in FHB resistance in winter wheat grown in controlled environment integrating SB are reduced largely to genotypic variations without ignoring the possibilities for physiological disorders, developmental errors, and environmental internal factors of the plants. Hence, when applying the protocol, the phenotypic evaluation results for instance of FHB results are repeatable once the standardized controlled environment of plant growth is met.

Over 500 genotypes from a breeding program and genebank were evaluated using the proposed protocol, and a good phenotypic diversity was observed in the studied germplasm. Moderate to high broad-sense heritability estimates were obtained based on replication in time for heading (0.69–0.79), FHB (0.53–0.57), FLA (0.41–0.53), spike length (0.70–0.77), and spike width (0.44–0.64). In previous studies, the average broad-sense heritabilities for FHB resistance traits were 0.54–0.73 (*sd*=0.15–0.18) based on field trials (Ma et al., 2020). The heritabilities in this work compared to previously published work indicate that FHB resistance is a moderately to highly heritable trait.

Fusarium head blight resistance is quantitatively inherited and controlled by a plethora of genes (Mesterhazy, 1995; Mesterhazy et al., 1999; Miedaner et al., 2010, 2019; Venske et al., 2019). In this work, the AUDPC showed that both highly resistant and susceptible genotypes were present in Nordic winter wheat. On average, the genebank germplasm was less susceptible to FHB than the breeding lines (Figure 1). This can be explained by the presence of some highly resistant germplasm in the genebank collection. Previous studies indicated that genetic resources, such as landraces, might harbor more resistance genes than elite lines (Kidane et al., 2017; Buerstmayr et al., 2020). The genetic variation for FHB resistance in the materials evaluated can be exploited to improve FHB resistance in the Nordic winter wheat.

In addition, we found high genetic variation for the heading stage, anthesis, spike length, spike width, and FLA. Similarly, high genetic variation for heading (Zanke et al., 2014), anthesis (Bogard et al., 2011), spike length (Zhai et al., 2016), and FLA (Liu et al., 2017, 2018) has been reported in winter wheat. These traits are important for agronomic adaptation and can have pleiotropic effects on disease severity, which may delay the use of resistance alleles in commercial cultivars (Gervais et al., 2002; Buerstmayr et al., 2020; Ogrodowicz et al., 2020). However, in this present study, we found very weak correlations between AUDPC (FHB) and all five agronomic traits measured. A high correlation between heading and anthesis is expected (Langer et al., 2014), since wheat ears usually emerge from the flag leaf before anthesis. However, in some cultivars, the ears may not fully emerge from the flag leaf before shedding pollens. Flag leaf is an important organ that influences yield-related traits, such as spike length, because of its role in photosynthesis and nutrient partitioning. The correlation between FLA and the two spike traits was low, only significant for spike length (Supplementary Figure 1). In earlier studies, Liu et al. (2018) also found a significant and positive correlation between spike length and flag leaf length.

Fusarium head blight resistance is quantitative, being controlled by many loci. The significant SNPs detected on chr. 3BS (62.31–68.71cM) might be associated with a major QTL (SLUfhbchr3B.4) that regulates FHB severity in the material analyzed (*p*=0.0001, Table 2; Supplementary Table 2). Within ±20cM, SLUfhbchr3B.4 overlapped with QTLs projected into meta-QTL 3/3B and 4/3B in the previous studies (Venske et al., 2019). The high impact *Fhb1* QTL originating from the Chinese spring wheat, Sumai 3, is located on the short arm of chr. 3B between 1cM and 7cM (Bai et al., 1999; Waldron et al., 1999; Venske et al., 2019; Ma et al., 2020). At *p*=0.001, the significant SNPs found between 9cM and 14cM on chr. 3B within the breeding set was localized between the *Fhb*1 QTL and meta-QTL 1/3B (16.02–16.84cM) reported by Venske et al. (2019). Similar to the outcome of this study, previous studies found QTLs for FHB resistance on the other sub-genomes of bread wheat (Miedaner et al., 2010, 2019; Kollers et al., 2013; Venske et al., 2019). For example, the QTL on chr. 3A (SLUfhbchr3A.3; Table 2) colocalized with the meta-QTL1/3A located at 14.01–26.18cM (Venske et al., 2019). The average effect of the favorable QTL alleles for six SNPs detected by at least two GWAS models simultaneously could reduce FHB severity below the overall mean (Supplementary Figure 2; Table 2). Since 1999, over 500 QTLs scattered across all wheat sub-genomes and chromosomes have been reported for FHB resistance, the sub-genome B containing the largest number of the QTLs followed by A (Venske et al., 2019). Chromosome 3B can be described as a hot spot for FHB resistance because the majority of the FHB QTLs found in our study and literature was localized on this sub-genome (Liu et al., 2009; Venske et al., 2019; Ma et al., 2020; Table 1; Figure 1; Supplementary Table 2). Colocalization of two QTLs between heading and FHB severity might partly explain the significant negative correlation between FHB and heading (*r*=−0.16, *p*=0.001). FHB resistance QTLs may be population specific and QTLs with minor effects control FHB resistance and are difficult to detect in smaller populations. In this study, the presence of common FHB resistance QTL regions in both breeding and genebank sets increased the power to detect more QTLs in the combined set and even at a higher significance threshold (e.g., Bonferroni corrected threshold at *α*=0.05; Figure 6). Thus, higher gains should be expected from MAS for FHB resistance in wheat breeding programs when lines from both breeding and genebank materials are used.

The genetic architecture of heading, anthesis, SW, SL, and FLA is complex, being influenced by several QTLs (Supplementary Tables 3 and 4). Similar to our results, Langer et al. (2014) and Zanke et al. (2014) found many QTLs for heading time, majority was located on chromosome 5B. Also, QTLs were reported for anthesis (Bogard et al., 2011), spike characteristics (Zhou et al., 2017), and FLA (Liu et al., 2018). The presence of QTLs in similar genomic regions might explain the positive and moderate phenotypic correlations observed between the heading stage and anthesis (*r*=0.51, *p*<0.001) as well as FLA and SL (*r*=0.23, *p*=0.001).

In GWAS, large population sizes are required to detect QTLs with small effects and to reduce the Beavis effect (Beavis and Paterson, 1998; Xu, 2003). Consequently, at *p*=0.0001, we found more QTLs for GWAS incorporating lines from both genebank and breeding sets than GWAS within each source population separately. However, within genebank set or breeding set, several QTLs could be detected at a lower significant threshold (e.g., *p*=0.001), only a few were present at *p*<0.0001, depending on the trait (Supplementary Tables 2 and 4). For example, the FHB QTLs on chr. 3B (SLUfhbchr3B.4) and 3D (SLUfhbchr3D.5a and SLUfhbchr3D.5b) in breeding set and genebank set (Supplementary Table 2). The results found for analyses within individual sets showed that both common QTLs and partially different QTLs might regulate FHB resistance in the two populations. The presence of some common resistance QTLs in both breeding and genebank sets might have increased the power to detect more QTLs in the combined set and even at a higher significance threshold (e.g., Bonferroni corrected threshold at *α*=0.05; Figure 6). Higher gains should be expected from MAS for FHB resistance breeding when lines from both breeding and genebank populations are used. A strategy to incorporate QTL from the genebank set to the breeding set will lead to improved resistance to FHB in the germplasm of the breeding program.

## Conclusion

Speeding up of the generation cycle was achieved by integrating SB protocol in diverse winter wheat genotypes used in the improvement for Nordic winter wheat cultivars. Within this work frame, screening for disease resistance among the genotypes for FHB was evaluated in the assigned Nordic germplasm. A significant genetic variation could be found for FHB resistance and agronomic traits in Nordic wheat germplasm. The molecular mechanism of FHB resistance is very complex, governed by multiple loci. Resistant alleles were present in both LM and NG materials and can be harnessed to improve FHB resistance in winter wheat by genomics-assisted speed breeding.

Due to the prolonged nature of winter wheat growth requiring vernalization at every generation, conventional breeding programs have the potential to release new cultivars in 15years. Taking into account the period required for vernalization, the current protocol for disease resistance in wheat provides the potential for reducing the growth by 55 to 110days per generation. Therefore, a significant time saving up to 2–3years can be expected in trait introgression breeding programs using several generations of backcrossing and 1year in conventional SSD programs.

## Data Availability Statement

The original contributions presented in the study are included in the article/Supplementary Material, further inquiries can be directed to the corresponding author.

## Author Contributions

AC and TH conceived the study. AC, MZ, and FO planned the greenhouse experiments. TH developed the breeding population set. MZ, FL, MA, and FO performed the greenhouse experiments. MZ and DG analyzed the data and wrote the first draft. All authors contributed to the data interpretation and approved the final version of this manuscript.

## Conflict of Interest

The authors declare that the research was conducted in the absence of any commercial or financial relationships that could be construed as a potential conflict of interest.

## Publisher’s Note

All claims expressed in this article are solely those of the authors and do not necessarily represent those of their affiliated organizations, or those of the publisher, the editors and the reviewers. Any product that may be evaluated in this article, or claim that may be made by its manufacturer, is not guaranteed or endorsed by the publisher.

## Footnotes

^1^ http://www.traitgenetics.com/en/
