# Supplementary material for: Characterizing Winter Wheat Germplasm for Fusarium Head Blight Resistance Under Accelerated Growth Conditions
Source: Front Plant Sci. 2021 Aug 25;12:705006. doi: 10.3389/fpls.2021.705006 (PMC8425451; doi:10.3389/fpls.2021.705006)
Supplement: Supplementary file 2 [file Data_Sheet_2.docx]

| Supplementary Figure 1: Pearson correlation among various phenotypic traits |
| --- |
| 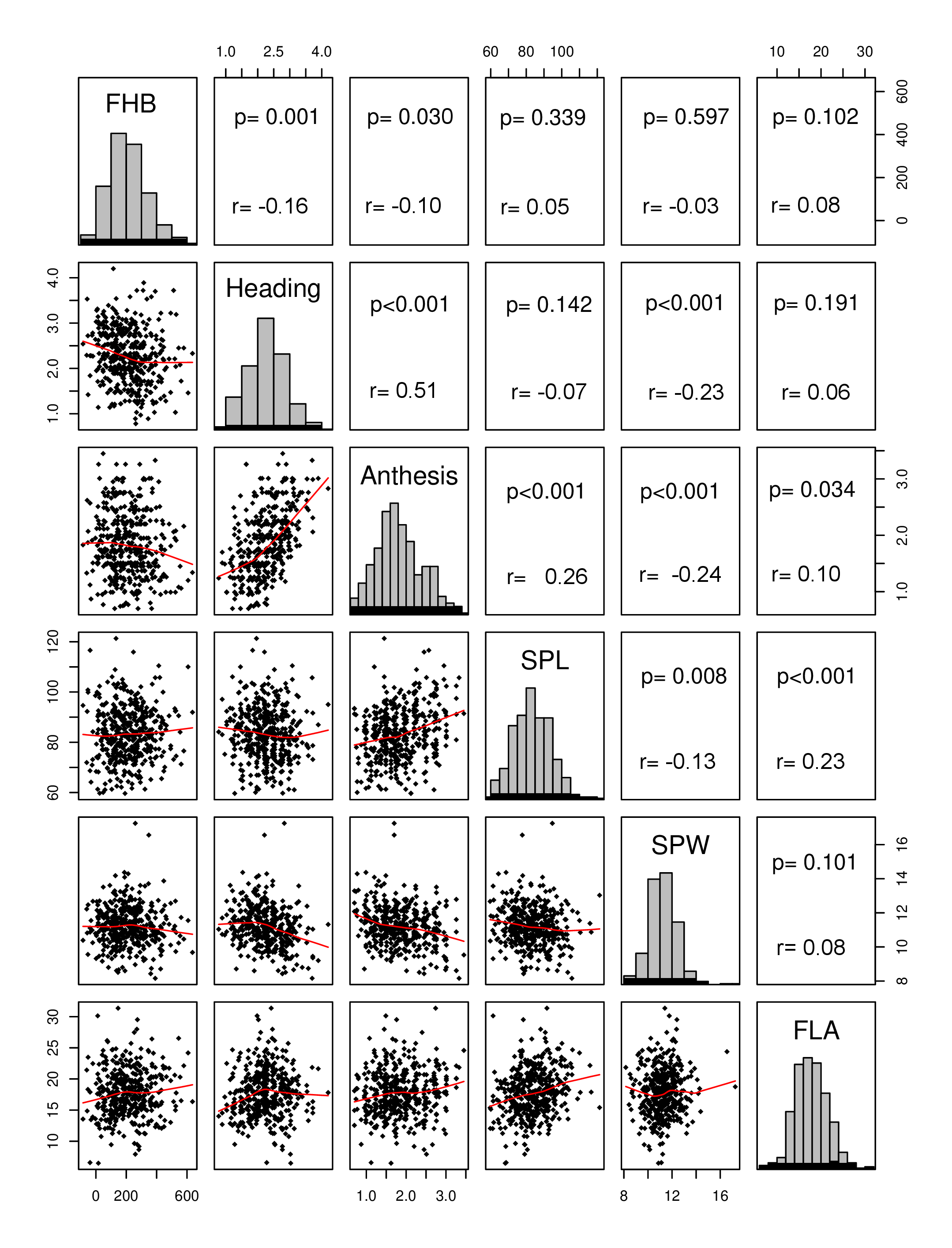 |

| Supplementary Figure 2: Average effect of the favorable QTL alleles for six SNPs detected by at least two GWAS models |
| --- |
|  |
